# The Development and Optimisation of a Spinosin Solid-Dispersion-Based Functional Dairy Beverage and Its Sleep-Promoting Effects in Mice

**DOI:** 10.3390/foods15010180

**Published:** 2026-01-05

**Authors:** Beizhi Zhang, Fuzhi Xie, Nannan Chen, Qing Zhang, Dan Zhao, Yu Chen, Shujing Xuan, Xiaona Liu, Liang Zhang

**Affiliations:** 1 National Center of Technology Innovation for Comprehensive Utilization of Saline-Alkali Land, Dongying 257000, China; zhangbeizhi777@163.com (B.Z.); xiefuzhi2002@163.com (F.X.); 2 Institute of Food Science and Technology, Chinese Academy of Agricultural Sciences, Beijing 100193, China; chennan0114@gmail.com (N.C.); 13345033761@163.com (Q.Z.); bszhaodan@163.com (D.Z.); cynthia1070@163.com (Y.C.); m14753632786@163.com (S.X.); 3 College of Food Science and Technology, Huazhong Agricultural University, Wuhan 430070, China; 4 College of Food Science and Engineering, Qingdao Agricultural University, Qingdao 266000, China; 5 Service Center for Comprehensive Utilization of Saline-Alkali Land in Agricultural High-Tech Industrial Demonstration Zone of the Yellow River Delta, Dongying 257000, China; hsjngqkjcxj@shandong.cn

**Keywords:** jujube seeds, spinosin, solid dispersion, functional dairy beverage, sleep aid

## Abstract

Insomnia remains a widespread global health issue, and traditional hypnotic drugs often produce adverse effects. Although spinosin in Ziziphi Spinosae Semen has sleep-promoting effects, its use is limited by poor solubility and low oral bioavailability. In this study, the solvent melt method was used to prepare spinosin solid dispersions, optimising the process with an L_9_(3^4^) orthogonal design based on apparent solubility. In vitro dissolution testing showed that solid dispersions of varying particle sizes dissolved more readily than pure spinosin, with smaller particles exhibiting faster dissolution. Cellular uptake was assessed in human colon adenocarcinoma cells, with results revealing enhanced uptake of smaller-particle solid dispersions. Powder X-ray diffraction confirmed that spinosin transformed from a crystalline to an amorphous state in the dispersion system. A quadratic orthogonal experiment was conducted to optimise functional dairy beverage formulation, using the centrifugal sedimentation rate as the evaluation index. In vivo experiments demonstrated that the resulting functional dairy beverage reduced spontaneous activity in mice, achieved a 60% sleep-onset rate, improved ethanol-induced memory impairment and produced marked sleep-promoting effects. Moreover, pharmacokinetic studies confirmed that the spinosin solid-dispersion-based functional dairy beverage significantly enhanced the systemic exposure and oral bioavailability of spinosin compared to the spinosin water suspension. These findings indicate that solid dispersion technology effectively enhances spinosin solubility and that the developed functional dairy beverage shows promise as a sleep-promoting functional food.

## 1. Introduction

Insomnia has become an increasingly severe global issue, with its high prevalence and substantial impact on public health representing a major global challenge [1,2]. Epidemiological evidence indicates that approximately 10% of adults worldwide experience chronic insomnia, whereas up to 20% suffer intermittent insomnia [3]. Insomnia is characterised by difficulty initiating and maintaining sleep, early awakening and strong associations with chronic conditions, such as depression, anxiety disorders and cardiovascular disease [4]. Persistent insomnia markedly reduces quality of life and imposes a considerable socioeconomic burden [5]. Clinically, treatments mainly comprise pharmacological and non-pharmacological approaches [6]. Although drugs can provide short-term relief, they often cause adverse effects, including dependence, tolerance and cognitive impairment [7,8]. In recent years, traditional Chinese medicine has shown distinct advantages in treating insomnia; among these, Ziziphi Spinosae Semen has garnered attention for its established sedative and sleep-promoting properties [9,10].

**Figure 1:**
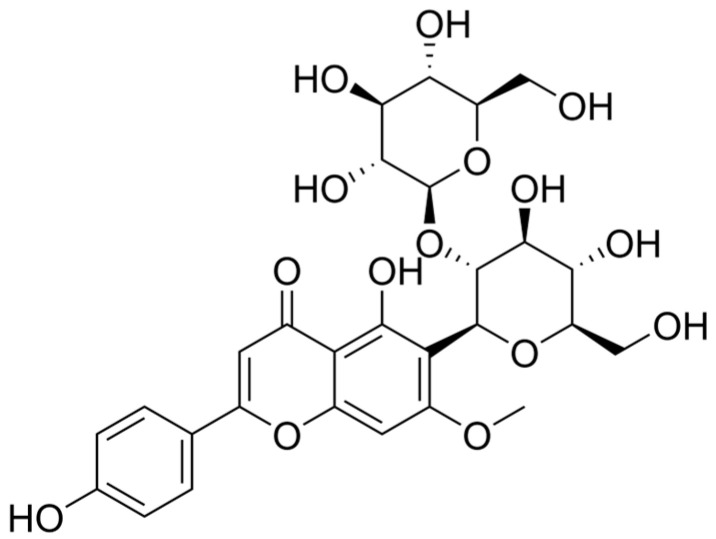
Chemical structure of spinosin.

One of the principal active components of Ziziphi Spinosae Semen, spinosin (molecular weight: 608.5 g/mol), is considered the key pharmacological constituent responsible for its sleep-enhancing effects [11,12,13,14]. Spinosin is a natural flavone-C-glycoside found mainly in the dried ripe seeds of *Ziziphus jujuba* Mill. var. *spinosa* (C_28_H_32_O_15_), belonging to the flavonoid glycoside class [15]; its chemical structure is shown in Figure 1. Reported solubility data indicate that spinosin is practically insoluble in water and ethanol [16]. Its content in raw Ziziphi Spinosae Semen typically ranges from ~0.44 to 1.71 mg/g of dried material [17].

Numerous studies have reported the pharmacological effects of spinosin [18], including hypnotic activity [19], cognitive improvement, anti-anxiety properties [20] and antioxidant effects [21]. Animal studies have consistently shown that spinosin markedly extends non-rapid eye movement (NREM) sleep and improves overall sleep structure. In 2025, Zhao et al. [22] found that spinosin substantially increased NREM sleep duration in mice by activating the gamma-aminobutyric acid (GABA) neurons in the nucleus accumbens and inhibiting orexin neurons in the lateral hypothalamus. Zhang et al. [23] further reported that spinosin (15 mg/kg) reduced c-Fos protein expression levels in the lateral hypothalamus and locus coeruleus, suggesting that it produces sedative and hypnotic effects via the inhibition of wake-promoting neural circuits. Ying et al. [24] also showed that a modified Ziziphi Spinosae Decoction enriched with spinosin improved sleep in insomnia mouse models, likely through modulation of the orexin system. Overall, spinosin appears to promote NREM sleep and enhance sleep quality by regulating neuronal activity in key brain regions, especially by strengthening GABAergic inhibition and orexin-mediated wakefulness. Therefore, spinosin holds promise for developing new sleep-promoting agents and functional foods.

Despite the notable biological activities of natural products, such as spinosin [18], their poor water solubility and low oral bioavailability severely restrict clinical translation and product development [16,25,26,27,28]. Solid dispersion technology, which is relatively simple to prepare, has become a commonly used strategy to improve the solubility of poorly soluble constituents in traditional Chinese medicine [29,30,31,32,33]. It is widely regarded as an effective means to enhance dissolution and bioavailability [34]. By dispersing poorly soluble drugs uniformly in hydrophilic carriers, solid dispersions increase wettability and specific surface area, thereby promoting dissolution and gastrointestinal absorption. Previous research has demonstrated the improved bioavailability of various natural components, including lycopene [35] and curcumin [36]. Jing et al. [37] enhanced resveratrol solubility and Caco-2 cell permeability using phytoglycogen–resveratrol solid dispersions prepared via co-solvent mixing and spray-drying. Resveratrol amorphous solid dispersions with polyvinylpyrrolidone or carboxymethyl chitosan have also been optimised to improve dissolution and stability, suggesting value for nutraceutical applications [38]. Similar solid dispersion approaches have been reported for epigallocatechin gallate and quercetin to address low solubility and bioavailability [39]. However, most studies have focused on physicochemical improvements, such as solubility, stability and cellular uptake, whereas few have translated solid dispersion systems into functional foods with validated safety and physiological efficacy.

The increasing emphasis on public health has driven consumer demand for functional foods and beverages with targeted health benefits, including sleep support. Dairy products, owing to their broad acceptability, favourable sensory profile and strong compatibility with active ingredients, are considered suitable carriers for functional beverages [40]. Building on this, our study addresses the existing gap by (i) developing a spinosin solid dispersion using food-grade excipients, (ii) incorporating it into a dairy beverage prototype and (iii) evaluating its safety and sleep-promoting effects in vivo. Specifically, this study applies solid dispersion technology to overcome limitations of poor solubility and low bioavailability in natural products and establishes a novel natural-product-based solid dispersion dairy beverage, providing a feasible route for sleep disorder intervention.

## 2. Materials and Methods

### 2.1. Materials

#### 2.1.1. Reagents and Chemicals

The main raw materials used in the study included spinosin (purity ≥ 98%; Shanghai Yueli Biotechnology Co., Ltd., Shanghai, China), poloxamer 188 (Beijing Solarbio Science & Technology Co., Ltd., Beijing, China), full-fat whole milk (Inner Mongolia Yili Industrial Group Co., Ltd., Hohhot, China), sucrose [purity ≥ 99.7%; Shanghai Sugar, Tobacco and Alcohol (Group) Co., Ltd., Shanghai, China], sodium carboxymethyl cellulose (CMC-Na; Beijing Chemical Reagent Co., Ltd., Sinopharm Group, Beijing, China), absolute ethanol (Beijing Chemical Reagent Co., Ltd., Beijing, China) and pentobarbital sodium (purity ≥ 98%; Beijing Ouchu Technology Co., Ltd., Beijing, China).

#### 2.1.2. Instruments

The following instruments were used in the study: a 36 V AC power supply, a high-speed refrigerated centrifuge (MGL-16MT, Shanghai Merick Instruments Co., Ltd., Shanghai, China), a 0.45-μm microporous membrane, a high-performance liquid chromatograph (Agilent 1200SL, Agilent Technologies, Inc., Santa Clara, USA), dialysis bags (molecular weight: 7000–14,000 Da; 25 × 16 mm) and a dynamic light scattering (DLS) analyser (Malvern Zetasizer Nano ZS, Malvern Instruments, Malvern, UK).

#### 2.1.3. Experimental Animals

Kunming (KM) mice (18–22 g; equal numbers of males and females) and Sprague-Dawley (SD) rats (180 ± 20 g; all male) were purchased from Sibeifu (Beijing) Biotechnology Co., Ltd., Beijing, China. All animals were housed in a specific pathogen-free level barrier facility at 22 °C ± 2 °C, 50–60% relative humidity and a 12/12 h light/dark cycle, with free access to standard feed and water.

### 2.2. Preparation of Spinosin Solid Dispersions

Spinosin solid dispersions were produced using the solvent-melting method. Spinosin and poloxamer 188 were weighed precisely according to preset mass ratios. Spinosin was dissolved completely in an appropriate volume of anhydrous ethanol. Poloxamer 188 was melted in a thermostatic water bath at the designated temperature, after which the spinosin solution was added slowly. The mixture was stirred at controlled speeds until full ethanol evaporation. The system was then transferred to a preset low-temperature environment and solidified for 6 h. The solidification product was dried, milled and sieved through an 80-mesh sieve. The resulting powder was stored in a light-protected desiccator.

### 2.3. Single-Factor and Orthogonal Experimental Design for Solid Dispersion Preparation

The effects of four key variables, namely the spinosin-to-poloxamer 188 mass ratio, melting temperature, stirring speed and cooling temperature, on the apparent solubility of the solid dispersions were assessed through single-factor experiments (Table 1). Each variable was tested while the remaining parameters were held constant to determine individual effects on apparent solubility:

(1) $$\mathrm{Apparent}\;\mathrm{solubility}\;(\%)=\frac{m_{0}}{m_{1\;}}\times 100,$$

where m_0_ represents the mass of dissolved spinosin and m_1_ is the total mass of the spinosin solid dispersion.

Based on the single-factor results, three levels within the optimal range were selected for each variable for refinement (Table 2). An L_9_(3^4^) orthogonal array was then applied to optimise the formulation, with apparent solubility as the primary response variable. Spinosin concentration was quantified using high-performance liquid chromatography. This design enabled efficient optimisation with minimal experimental runs while maintaining statistical reliability.

### 2.4. Characterisation and Property Evaluation of Solid Dispersions

#### 2.4.1. Particle Size Characterisation

Five milligrams of solid dispersion powder was dispersed in 10 mL of deionised water (pH 7.4). Samples were sonicated for 5, 25 and 45 min to ensure uniform dispersion. A DLS analyser was employed to measure the average hydrated particle size (Z-average) of the redispersed nanoparticles/micelles at 25 °C. Each sample was tested in triplicate, and the mean value was recorded.

#### 2.4.2. In Vitro Dissolution Testing

A solid dispersion sample, equivalent to 30 mg of spinosin, was suspended in 2 mL of simulated intestinal fluid (SIF; sterile, pH 6.8) and transferred into a pre-treated dialysis bag (molecular weight cut-off: 7000–14,000 Da) [41,42]. The release medium consisted of 900 mL of ultrasonically degassed SIF, maintained at 37 °C and stirred at 100 rpm. Aliquots (3 mL) were collected at fixed intervals and replaced immediately with fresh medium to maintain the dissolution system at a constant total volume. After filtration through a 0.45 μm membrane, spinosin concentrations were determined using high-performance liquid chromatography, and dissolution curves were plotted.

(2) $$\mathrm{Cumulative}\;\mathrm{dissolution}\;\left(\%\right)=\frac{C_{n}\cdot V+\sum_{i=1}^{n-1}C_{i}\cdot V_{s}}{m_{2}}\times 100,$$

where C_*n*_ is the concentration of spinosin at time *n*, V is the total dissolution medium volume (900 mL), V_*s*_ is the sampling volume (3 mL), $\sum_{i=1}^{n-1}C_{i}\cdot V_{s}$ is the cumulative dissolution amount from previous samples and m_2_ is the initial drug loading (30 mg).

#### 2.4.3. Assessment of Cell Uptake Capacity

Partial human colon adenocarcinoma (Caco-2) cells were co-incubated with spinosin solid dispersions from Ziziphi Spinosae Semen of varying particle sizes for 1 h. After incubation, the cells were rinsed with phosphate-buffered saline to eliminate residual particles and subsequently fixed using 4% paraformaldehyde. The cell membranes and cytoskeletons were stained with fluorescent green phalloidin, whereas nuclei were counterstained with 4′,6-diamidino-2-phenylindole (i.e., DAPI). Near-infrared fluorescence from the spinosin solid dispersions was detected using a Zeiss AxioObserver inverted microscope (Carl Zeiss Microscopy GmbH, Jena, Germany) equipped with a near-infrared filter. Following image capture, the three fluorescence channels from the same field were merged in ImageJ 1.54g to visualise intracellular distribution of the Ziziphi Spinosae Semen spinosin solid dispersions and assess differences in cellular uptake among formulations with distinct particle sizes.

#### 2.4.4. Powder X-Ray Diffraction Characterisation of the Spinosin Solid Dispersion System

The crystalline characteristics of spinosin, poloxamer 188 and the combined spinosin–poloxamer 188 solid dispersion were examined via powder X-ray diffraction (PXRD). Measurements were obtained using a diffractometer with Cu Kα radiation operated at 40 kV and 40 mA. Each sample was lightly levelled in the holder and scanned at room temperature across a 2θ range of 3–45°, using a 0.02° step size and a 8°/min scan rate [43].

### 2.5. Solid Dispersion Emulsion Beverage Preparation Method

Spinosin solid dispersion, white granulated sugar and CMC-Na were accurately weighed, and full-fat pure milk was measured using a pipette. All components and the milk were transferred to a 100 mL volumetric flask and diluted to volume with distilled water. The mixture was continuously stirred on a magnetic stirrer at an appropriate speed until fully dissolved and homogeneous, producing the functional dairy beverage sample solution. An appropriate sample was placed into a centrifuge tube and centrifuged in a high-speed refrigerated centrifuge under preset conditions. The sedimentation rate was recorded to assess how different factor levels influenced the physical stability of the functional dairy beverage.

### 2.6. Single-Factor and Orthogonal Experimental Design for Preparation of the Solid Dispersion Emulsion Beverage

The influence of key formulation parameters for the spinosin solid dispersion dairy beverage, namely the solid dispersion mass fraction, milk volume, sucrose mass fraction and CMC-Na mass fraction, on the centrifugal precipitation rate was examined through single-factor trials. For each parameter, the remaining variables were kept constant to isolate specific effects on the centrifugal precipitation rate (Table 3):

(3) $$\mathrm{Centrifugal}\;\mathrm{precipitation}\;\mathrm{rate}\;(\%)=\frac{m_{3}}{m_{3}{+m}_{4}}\times 100,$$

where m_3_ represents the constant weight of the sediment dried at 105 °C after centrifugation and m_4_ denotes the constant weight of the supernatant dried at 105 °C.

Based on the single-factor findings, three levels within the optimal ranges were selected for each of the four factors to refine the formulation window (Table 4). An L_9_(3^4^) orthogonal array design was employed to optimise the dairy beverage formulation, using the centrifugal precipitation rate as the primary evaluation index.

### 2.7. Assessment of Sleep-Promoting Efficacy

Because the spinosin solid dispersion constitutes a newly developed active system, preliminary testing in mice was required to confirm safety, ethical suitability and functional plausibility before any potential human studies [33,44].

In total, 80 KM mice were randomly allocated into four groups (*n* = 20 each). Group A served as the control (distilled water), Group B received the spinosin solid-dispersion-based functional dairy beverage, Group C received an aqueous spinosin solution prepared with distilled water and Group D received pure milk without spinosin. Wang et al. [45] reported that spinosin enhances pentobarbital-induced hypnosis in a dose-dependent manner, with 10 mg/kg shown to produce sleep-promoting effects without additional agents. The treatment groups were orally administered the corresponding preparations at 10 mg/kg (calculated as spinosin) by gavage (20 mL/kg dosage volume) once daily for 14 days; the control group received the same volume of distilled water. After the final administration, subsequent behavioural or biological sample collection was performed.

#### 2.7.1. Ethanol-Induced Memory Retrieval Impairment Experiment in Mice: Step-Down Assay

Twenty-four hours before the last intragastric administration, mice were placed individually in a step-down apparatus and allowed to adapt for 3 min. A 36 V alternating current stimulus was then applied, and each mouse underwent 5 min of training. After a further 24 h, testing began: mice received the appropriate functional dairy beverage or distilled water by gavage, followed 30 min later by a 54% ethanol solution at 0.2 mL/20 g body weight. After a further 10 min, each mouse was placed onto the step-down platform and timed immediately. The latency to the first step-down and the number of incorrect responses (i.e., step-downs) within 5 min were recorded. These two measures were used as indicators of memory retrieval. Differences in latency and error frequency among the groups were compared to assess the extent to which each intervention mitigated ethanol-induced memory impairment.

#### 2.7.2. Self-Activity Ability Experiment

Thirty minutes after the final gavage, mice were placed individually into the open field apparatus designed for KM mice, with one animal per box. After 3 min of acclimation, spontaneous activity was recorded continuously for 10 min. This measure reflected central excitability and served as the evaluation parameter for spontaneous locomotor activity. Differences in the number of activities among the groups were compared to determine how each intervention influenced the animals’ spontaneous behaviour.

#### 2.7.3. Subhypnotic Sodium Pentobarbital Dosing Experiment

One hour after the final intragastric administration, mice in each group were injected intraperitoneally with sodium pentobarbital at 32 mg/kg. This dose had been identified as subhypnotic in preliminary tests conducted prior to the formal study. In this pre-experiment, a series of pentobarbital sodium doses (20–36 mg/kg, intraperitoneally administered) were assessed in KM mice to determine a level that induced sleep (defined as loss of the righting reflex for ≥1 min) in fewer than 10% of animals following the pharmacological criterion described by Wang et al. [18],. The 32 mg/kg dose consistently produced a sleep-onset rate below 10%, confirming its suitability for subhypnotic sleep-potentiation assessments.

After the administration of the subhypnotic dose, the number of mice that fell asleep in each group was recorded, and the sleep rate was calculated. Statistical comparisons were then performed to assess between-group differences.

### 2.8. Pharmacokinetic Study of Spinosin and Its Solid Dispersion Dairy Beverage

Appropriate quantities of spinosin powder and the spinosin solid-dispersion-based functional dairy beverage were dispersed in normal saline to prepare suspensions at 8 mg/mL (calculated as spinosin), designated as “spinosin water suspension” (SP-W) and “spinosin solid dispersion dairy suspension” (SP-D), respectively. Each suspension was vortex-mixed thoroughly to ensure uniformity and stability.

Twelve healthy SD rats were randomly allocated to two groups (*n* = 6 per group). Before dosing, rats were acclimated to laboratory conditions for 1 day, with free access to water but fasted for 12 h. Animals were then given SP-W or SP-D orally at 16 mg/kg (spinosin-equivalent), with gavage volume adjusted to body weight. Blood samples (~0.3 mL) were collected via retro-orbital puncture at 0.5, 1, 2, 3, 5, 7, 9, 12, 18, 24, 36 and 48 h post-dose. Plasma was obtained via centrifugation at 4000 rpm and low temperature for 2 min, and supernatants were stored at −20 °C until analysis [46].

For plasma processing, 100 μL of plasma was transferred to a centrifuge tube, mixed with 1.5 mL of acetonitrile, vortexed for 3 min and centrifuged at 4000 rpm for 2 min. The organic supernatant was collected and evaporated to dryness under a gentle nitrogen stream. The residue was reconstituted in 100 μL of the mobile phase, vortexed for 6 min and filtered through a 0.22-μm membrane before high-performance liquid chromatography analysis.

Chromatographic conditions were as follows: Agilent C18 column (250 mm × 4.6 mm, 5 μm); mobile phase: acetonitrile–0.15% phosphoric acid aqueous solution (40:60, *v*/*v*); detection wavelength: 336 nm; column temperature: 35 °C; flow rate: 1.0 mL/min; injection volume: 20 μL. Spinosin concentrations in rat plasma were quantified using an external standard calibration method, after which pharmacokinetic parameters were calculated [47].

### 2.9. Statistical Analysis

All values are presented as means ± standard deviations. Data were analysed using Origin 2024 and SPSS 26.0 statistical software. Statistical significance was set at *p* < 0.05 and determined using Duncan’s multiple range test.

## 3. Results

### 3.1. Analysis of Single-Factor Experiments on Solid Dispersion Preparation

**Figure 2:**
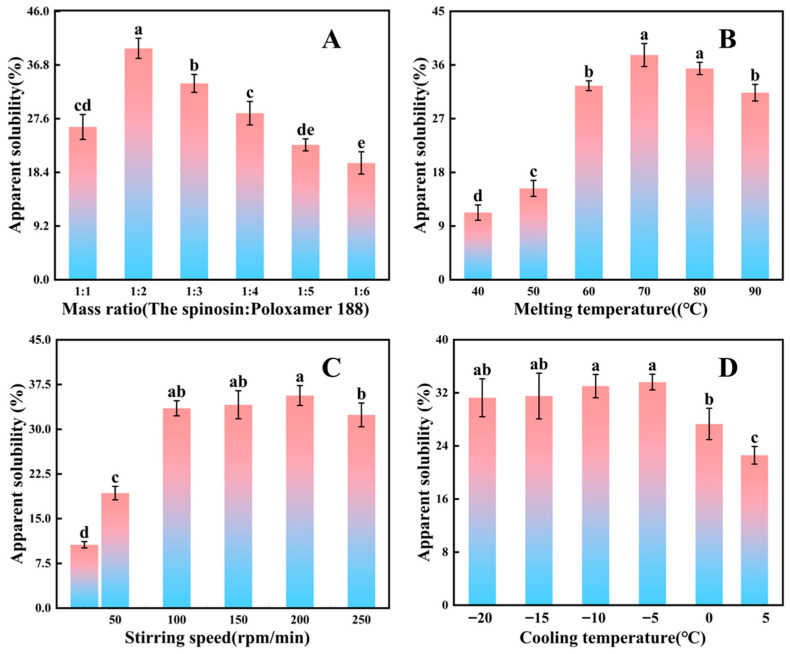
Effects of different factors on the apparent solubility of spinosin solid dispersion from jujube seeds. (**A**) Mass ratio, (**B**) melting temperature, (**C**) stirring speed and (**D**) cooling temperature. Data are means ± standard deviations (*n* = 3). Different letters indicate significant differences (*p* < 0.05).

According to the experimental data (Figure 2A), when the mass ratio of spinosin to poloxamer was 1:2, spinosin’s apparent solubility reached its highest value of 39.66% ± 1.72%, which was significantly higher than that of the other ratio groups. Regarding the carrier’s mechanism, poloxamer, a water-soluble polymer, contains polyethylene oxide and polypropylene oxide chains that interact with spinosin via hydrogen bonds to form intermolecular complexes. When the poloxamer proportion was too low (e.g., 1:1), the carrier could not fully encapsulate spinosin, leaving drug molecules as microcrystals and limiting dissolution. However, when the mass ratio rose to ≥1:3, apparent solubility declined. This decrease likely reflects reduced drug loading in the solid dispersion [48], and excessive poloxamer may also form micellar aggregates in solution, restricting drug diffusion and lowering the amount of spinosin dissolved per unit volume [49].

Experimental results (Figure 2B) showed that as the melting temperature increased from 40 °C to 70 °C, the apparent solubility of spinosin rose significantly, reaching a maximum of 37.68% ± 1.93% at 70 °C, following a ‘first increase then decrease’ pattern. From a thermodynamic perspective, higher temperature improves spinosin solubility in the molten carrier and accelerates evaporation of anhydrous ethanol [50], reducing residual solvent effects on drug dispersion. Excessively high temperatures may cause two issues: first, as a flavonoid, spinosin may undergo partial oxidation and degradation after prolonged heating, decreasing active ingredient content and apparent solubility; second, high temperatures may induce thermal ageing of poloxamer chains [51], weakening their interaction with the drug and destabilising the dispersion, which promotes recrystallisation and ultimately reduces solubility.

As shown in Figure 2C, the apparent solubility of spinosin increased overall as stirring speed rose from 25 to 200 rpm, reaching a peak value of 35.66% ± 1.67% at 200 rpm. However, when the speed was further increased to 250 rpm, solubility declined. At low stirring speeds, inadequate agitation causes uneven mixing of the spinosin ethanol solution with molten poloxamer, producing a ‘layering’ effect in which the drug accumulates in aggregates that dissolve poorly. As the speed increases, the enhanced shear force disrupts the aggregates and promotes uniform dispersion of spinosin in the molten carrier [52,53]. Simultaneously, the faster evaporation of anhydrous ethanol lessens residual solvent interference with drug dispersion and dissolution, thereby improving solubility.

Experimental results (Figure 2D) also showed that as the cooling temperature decreased from 4 °C to −5 °C, the apparent solubility of spinosin increased significantly, reaching 33.62% ± 1.19% at −5 °C. When the temperature further decreased to −10 °C, −15 °C or −20 °C, solubility remained relatively stable without a notable upwards shift. Once the temperature falls below −5 °C, although the cooling rate increases, the phase transition between the drug and the carrier is largely complete, and the drug’s physical state (mainly amorphous or microcrystalline) remains stable [54,55], leading to stabilised solubility. Additionally, excessively low cooling temperatures (e.g., −20 °C) increase energy consumption and processing costs while making the solid dispersion more brittle, affecting subsequent grinding and sieving.

### 3.2. Orthogonal Experiment Analysis of Solid Dispersion Preparation

Based on the single-factor experiment results, the mass ratio of spinosin to poloxamer, melting temperature, stirring speed and cooling temperature were selected as optimisation variables, and an L_9_(3^4^) orthogonal design was constructed with four factors at three levels. The apparent solubility of the spinosin solid dispersion served as the response index. The statistical outcomes are shown in Table 5 (across experiments 1–9, apparent solubility ranged from 25.09% to 38.29%, with K, k and R values listed). The data indicated that the relative influence of each factor on solubility was, in decreasing order, mass ratio of spinosin to poloxamer (R = 6.77) > stirring speed (R = 3.41) > melting temperature (R = 3.32) > cooling temperature (R = 1.49).

Among these factors, the mass ratio of spinosin to poloxamer exerted the strongest significant effect on solubility (F = 24.01 > F_0.05_ = 19.00, *p* < 0.05) (Table 6). The optimal combination was identified as spinosin:poloxamer = 1:2 (A_2_), melting temperature = 70 °C (B_2_), stirring speed = 200 rpm (C_3_) and cooling temperature = −15 °C (D_3_), namely A_2_B_2_C_3_D_3_. All subsequent validation experiments were conducted under these conditions.

### 3.3. Characterisation of Spinosin Solid Dispersion Redispersion Particle Sizes

**Figure 3:**
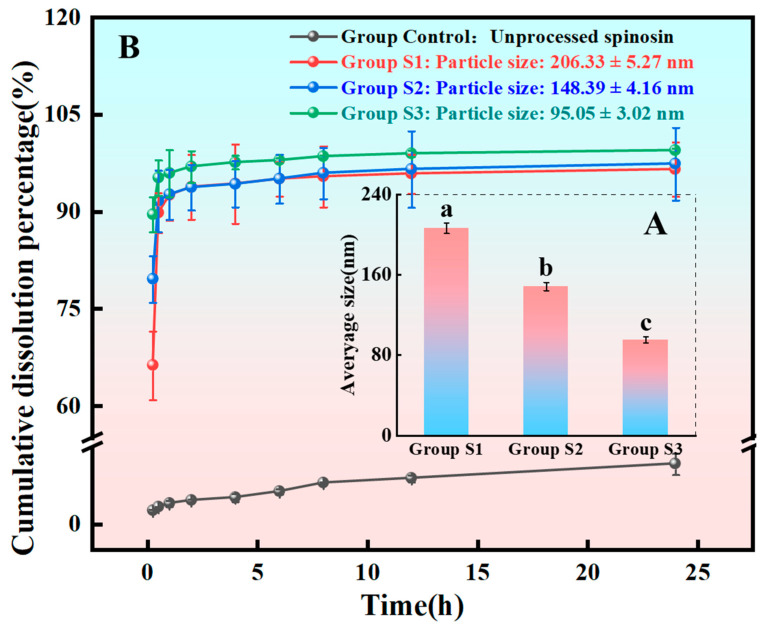
(**A**) Particle size distribution of three spinosin solid dispersion samples (*n* = 3). Different letters indicate significant differences among groups (*p* < 0.05). (**B**) Cumulative dissolution percentage of dispersions with different particle sizes (*n* = 3).

Three batches of spinosin solid dispersions with different redispersion particle sizes were produced by varying ultrasonic time; the mean hydrated particle sizes measured via DLS were 206.33 ± 5.27 nm (group S1), 148.39 ± 4.16 nm (group S2) and 95.05 ± 3.02 nm (group S3) (Figure 3A). Particle size distributions were uniform, and batch reproducibility was high. Thus, particle size in aqueous media could be reliably controlled by fine-tuning solvent melt parameters, enabling nanoscale optimisation. Moreover, the prepared solid dispersions with defined particle sizes provide a robust experimental basis for evaluating how particle size alters dissolution behaviour.

### 3.4. Analysis of in Vitro Dissolution Test Results

The use of pH 6.8 SIF as the dissolution medium more closely mimics small-intestinal conditions, increasing the predictive accuracy of in vitro assessments for oral bioavailability. As shown in Figure 3B, all spinosin solid dispersions exhibited significantly improved dissolution compared with the unprocessed spinosin control, and a clear particle size dependence emerged: smaller particles produced faster and higher-level cumulative dissolution. Solid dispersion groups S1–S3 dissolved rapidly and almost completely within 2 h, in contrast to the poor dissolution of unprocessed spinosin. Notably, group S3 (95.05 ± 3.02 nm) achieved the fastest and greatest dissolution, demonstrating that smaller particle size enhances dissolution under intestinal conditions. These results show that nanosizing combined with solid dispersion formulation markedly improves spinosin solubility and intestinal availability [56].

### 3.5. Cellular Uptake Imaging Analysis of Spinosin Solid Dispersions with Varying Particle Sizes in Caco-2 Cells

**Figure 4:**
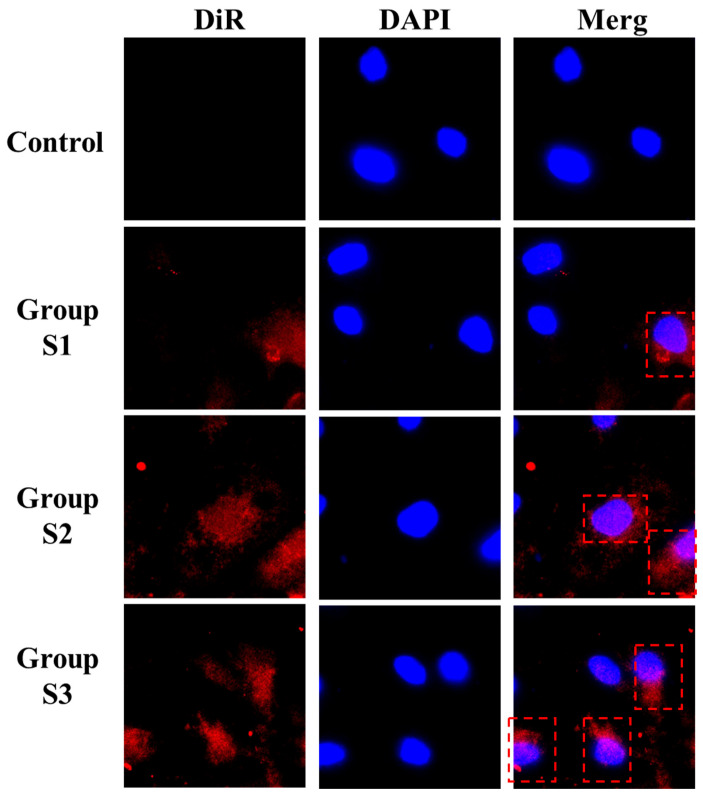
Uptake and distribution profiles of spinosin solid dispersions with varying particle sizes in human colon adenocarcinoma (Caco-2) cells. Control: unprocessed spinosin; Group S1: 206.33 ± 5.27 nm particle size; group S2: 148.39 ± 4.16 nm; group S3: 95.05 ± 3.02 nm.The red dashed boxes indicate representative regions showing intracellular uptake of spinosin solid dispersions.

In this experiment, Caco-2 cells were used to assess the uptake behaviour of DiR-labelled spinosin solid dispersions with varying particle sizes (groups S1–S3). Results revealed that no detectable near-infrared DiR fluorescence appeared in the control cells, excluding interference from endogenous and exogenous background signals and establishing a reliable experimental baseline (Figure 4). After incubating the cells with the three DiR-labelled solid dispersions with different particle sizes for 1 h and thoroughly washing to remove unbound particles, all treatment groups displayed significant fluorescence signals exceeding those of the control, and the fluorescence distribution was consistent with overall cell morphology, confirming that the signal derived from effective uptake of the particles. Notably, increased uptake of DiR-labelled solid dispersions resulted in enhanced intracellular fluorescence. Differences in fluorescence intensity were also apparent among the three particle size groups, with group S3 exhibiting the strongest signal and group S1 the weakest, suggesting that particle size strongly influences uptake efficiency, and that smaller particles favour cellular internalisation [57,58].

### 3.6. PXRD-Based Structural Analysis of the Spinosin–Poloxamer 188 Solid Dispersion System

**Figure 5:**
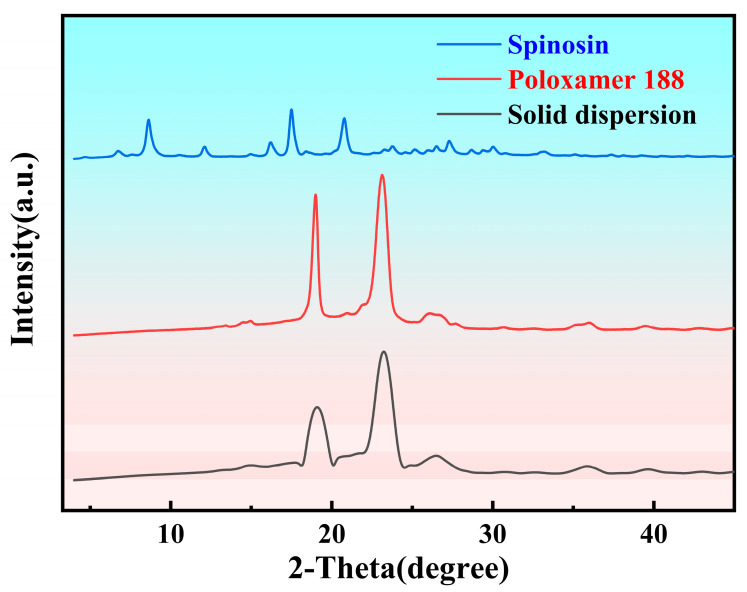
PXRD pattern of the spinosin solid dispersion system.

As shown in Figure 5, unprocessed spinosin exhibited distinct diffraction peaks at 2θ = 8.6°, 17.5° and 20.8°, confirming its crystalline nature. Poloxamer 188 showed broad reflections near 2θ ≈ 19° and 23° [59], consistent with its semi-crystalline structure. In contrast, the PXRD pattern of the solid dispersion showed the disappearance of the characteristic spinosin peaks, indicating that spinosin was fully incorporated into the polymer matrix in a non-crystalline form. These results confirm the conversion of crystalline spinosin into an amorphous state within the solid dispersion, qualitatively explaining the significant increase in solubility.

### 3.7. Analysis of Single-Factor Experiments on Spinosin Solid Dispersion Preparation in a Functional Dairy Beverage

**Figure 6:**
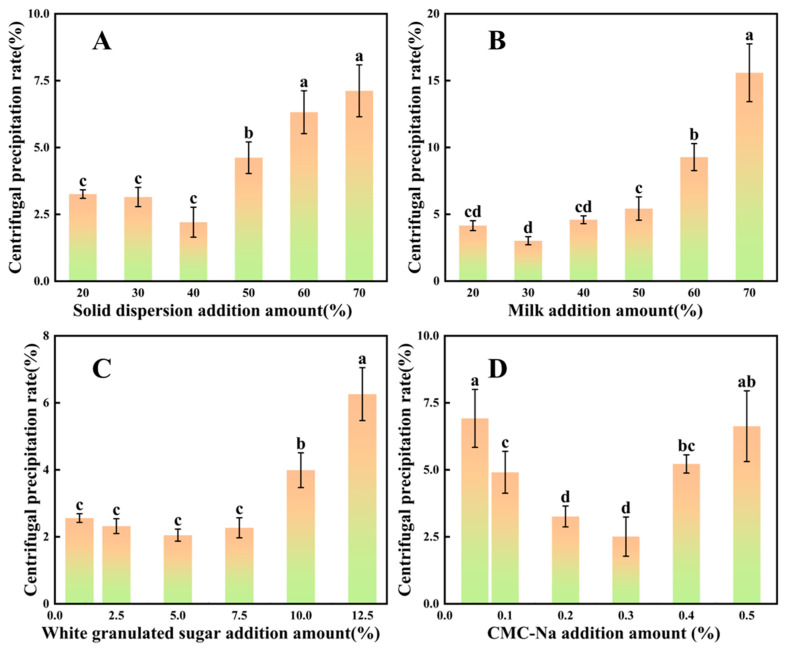
Influence of different additions on the centrifugal stability of spinosin solid dispersions in functional dairy beverages prepared from jujube seeds (*n* = 3). (**A**) Solid dispersion addition amount. (**B**) Milk addition amount. (**C**) White granulated sugar addition amount. (**D**) Sodium carboxymethyl cellulose (CMC-Na) addition amount. Different letters indicate significant differences (*p* < 0.05).

Experimental data (Figure 6A) showed that when solid dispersion content was 40%, the centrifugal sedimentation rate was lowest at 2.21% ± 0.56%. When the content was either below or above this level, the sedimentation rate rose. At lower contents, the dispersed-phase particle concentration was insufficient, the inter-particle spacing increased and weaker Brownian motion provided limited resistance to the gravitational force, resulting in faster sedimentation and a higher centrifugal sedimentation rate [60]. At 40% content, a moderate particle concentration enabled the formation of a stable three-dimensional colloidal network via combined electrostatic repulsion and steric hindrance, thereby reducing sedimentation and yielding the minimum sedimentation rate. However, when the solid dispersion content exceeded 40%, particle concentration became excessive, inter-particle spacing decreased and stronger van der Waals forces promoted particle aggregation to produce larger aggregates. These larger aggregates settled more readily under centrifugal force, markedly increasing sedimentation.

Figure 6B shows that the centrifugal sedimentation rate was lowest when milk content reached 30%. Below this level, the protein concentration was insufficient to fully coat the surface of solid dispersion particles, leaving exposed regions prone to direct contact and aggregation, thereby raising the sedimentation rate [61,62]. With milk content above 30%, excess protein molecules tended to self-associate (e.g., through casein micelle aggregation), forming large protein complexes that sedimented easily and also interacted with the solid dispersion particles, further disrupting system stability.

Experimental data (Figure 6C) indicated that when white granulated sugar content was 5%, the centrifugal sedimentation rate was lowest at 2.05% ± 0.18%. At contents below or above 5%, the sedimentation rate increased and reached 6.26% ± 0.79% at 12.5% content. From the perspective of osmotic pressure and particle dispersion, sucrose formed after dissolution increase the functional dairy beverage system’s osmotic pressure [63]. At 5%, the osmotic pressure of the system is moderate and balances the hydration film on the particle surface, stabilising the film and reducing particle aggregation. An appropriate sucrose concentration can also adsorb onto particle surfaces, enhancing electrostatic repulsion between particles and improving overall system stability.

CMC-Na, a commonly used food thickener and stabiliser, improved functional dairy beverage stability by increasing system viscosity and creating steric hindrance. As shown in Figure 6D, when CMC-Na content was 0.3%, the centrifugal sedimentation rate was lowest at 2.51% ± 0.73%. The carboxyl groups in CMC-Na dissociate in aqueous solution, generating negatively charged molecular chains [48]. Although this gel-like structure can hinder sedimentation, excessive CMC-Na molecular chains may over-adsorb onto the solid dispersion particles, reducing surface charge density and weakening electrostatic repulsion. This facilitates particle aggregation and the formation of large aggregates that sediment more readily.

### 3.8. Orthogonal Experiment Analysis of the Solid-Dispersed Functional Dairy Beverage

Based on the single-factor experiment results, the mass fraction of the spinosin solid dispersion, milk content, mass fraction of white granulated sugar and mass fraction of CMC-Na were selected as optimisation factors. An L_9_(3^4^) orthogonal experimental table was constructed, including these four factors at three levels (Table 7). The centrifugal sedimentation rate of the spinosin solid dispersion functional dairy beverage was used as the response value. Statistical analysis of the data is shown in Table 7 (sedimentation rates for tests 1–9 ranged from 2.05% to 7.35%, and K, k and R values are detailed). The order of factor influence on sedimentation was CMC-Na content (R = 2.86) > spinosin solid dispersion content (R = 2.16) > white granulated sugar content (R = 0.82) > milk content (R = 0.50).

As shown in Table 8, CMC-Na and spinosin solid dispersion contents were the main determinants of functional dairy beverage stability (F = 32.71 and 19.21, respectively; both >F_0.05_ = 19; *p* < 0.05). The optimal formulation was 40% spinosin solid dispersion, 20% milk, 5% white granulated sugar and 0.3% CMC-Na (i.e., A_2_B_1_C_2_D_3_). All subsequent experiments were conducted using this combination.

### 3.9. Sleep-Promoting Effects of Spinosin Solid-Dispersion-Based Functional Dairy Beverage in Mice

The sleep-promoting potential of the spinosin solid-dispersion-based functional dairy beverage was assessed via spontaneous activity, subthreshold pentobarbital-induced sleep and ethanol-induced memory impairment tests in mice. The spinosin solid-dispersion-based functional dairy beverage significantly reduced spontaneous locomotor activity, enhanced sleep induction under subthreshold hypnotic conditions and improved ethanol-impaired cognition. Compared with the nondispersed spinosin dairy beverage, the solid dispersion system markedly increased sedative, hypnotic and neuroprotective efficacy. The improved pharmacodynamic effects may be attributed to enhanced solubility, dispersion uniformity and intestinal absorption conferred by the solid dispersion formulation. Moreover, these results may support the development of spinosin-based functional foods or beverages with sleep-promoting activity.

#### 3.9.1. Ethanol-Induced Memory Impairment: Step-Down Assay

In the step-down passive avoidance test, control mice (group A) displayed short latency (35.66 ± 5.02 s) and a high error count (6.58 ± 1.01), indicating substantial memory impairment (Table 9). The spinosin solid-dispersion-based functional dairy beverage (group B) significantly improved performance, prolonging latency to 192.51 ± 3.99 s and reducing the error count to 1.69 ± 0.65 (*p* = 0.028). Compared with controls, group B extended latency by ~440% and reduced errors by ~74%, demonstrating robust cognitive protection against ethanol-induced dysfunction. The aqueous spinosin solution (group C) showed only marginal improvement (latency: 46.32 ± 4.80 s; error count: 5.33 ± 1.25), indicating spinosin’s limited efficacy without solid dispersion. The pure milk group (group D) showed no significant protective effect (latency: 37.24 ± 5.81 s; error count: 6.32 ± 0.78), confirming that the observed benefits in group B were attributable to the solid dispersion system rather than the dairy matrix.

#### 3.9.2. Subthreshold Pentobarbital-Induced Sleep Test

Following administration of a subthreshold pentobarbital dose, none of the control mice (group A) entered sleep, confirming that 32 mg/kg pentobarbital alone is insufficient to induce sleep (Table 9). The spinosin solid-dispersion-based functional dairy beverage (group B) substantially potentiated the hypnotic effect, with 12 of 20 mice falling asleep (60% sleep rate). The aqueous spinosin solution (group C) had minimal effect (10% sleep rate), whereas the pure milk group (group D) showed no sleep-inducing activity. Thus, the solid dispersion formulation markedly lowers the pentobarbital-induced sleep threshold, whereas free spinosin or milk alone exert negligible or no effects.

#### 3.9.3. Spontaneous Activity Test

As shown in Table 9, the control group (group A) exhibited a spontaneous activity of 315.62 ± 19.22 activities/10 min. The spinosin solid-dispersion-based functional dairy beverage (group B) significantly reduced locomotor activity to 195.96 ± 29.03 activities/10 min (*p* = 0.035), representing a ~38% decrease relative to the controls. In contrast, the aqueous spinosin solution (group C; 289.31 ± 31.58 activities/10 min) and pure milk (group D; 308.54 ± 17.49 activities/10 min) groups showed activity levels comparable to the controls, indicating that neither free spinosin nor the dairy matrix alone exerts a notable sedative effect. The similarity among groups A, C and D underscores the superior efficacy of the spinosin solid-dispersion-based functional dairy beverage, confirming that the solid dispersion system is essential for enhancing spinosin’s central inhibitory activity.

### 3.10. Pharmacokinetic Evaluation of Spinosin in SD Rats

**Figure 7:**
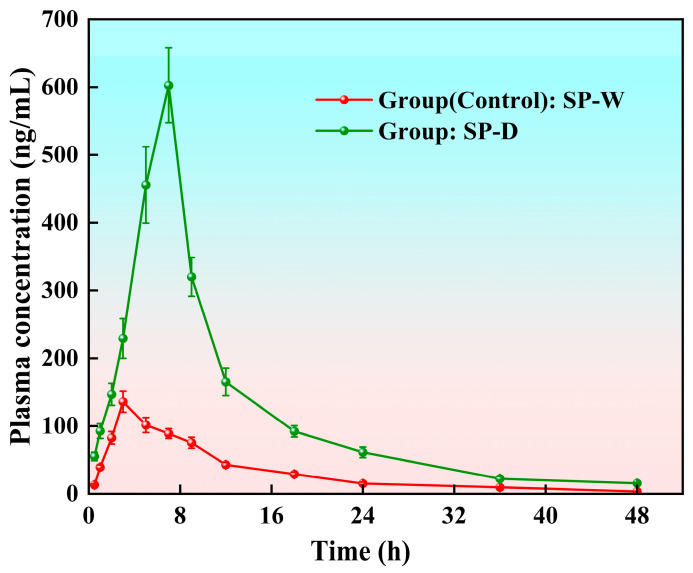
Plasma concentration–time profiles of spinosin in rats after oral administration of a spinosin water suspension (SP-W) and a spinosin solid-dispersion-based dairy suspension (SP-D).

Plasma concentration–time profiles (Figure 7) and pharmacokinetic parameters (Table 10) revealed that oral administration of the spinosin solid-dispersion-based functional dairy beverage (i.e., SP-D) substantially improved systemic exposure and absorption of spinosin compared with the spinosin water suspension (i.e., SP-W).

Following SP-W administration, spinosin exhibited a rapid absorption phase followed by a steep decline in plasma concentration, indicating limited systemic exposure and poor retention. In contrast, SP-D demonstrated a slower initial decline and sustained plasma levels over 48 h, reflecting enhanced absorption and extended circulation. The higher terminal-phase plasma concentrations of SP-D suggest improved solubilisation and bioavailability conferred by the solid dispersion system, likely facilitating more efficient gastrointestinal absorption and delayed elimination [64].

Pharmacokinetic parameters were calculated using a two-compartment model (Table 10). SP-D exhibited a significantly prolonged distribution half-life and elimination half-life, indicating that the dairy-based spinosin solid dispersion delayed distribution and extended systemic circulation. Notably, the maximum plasma concentration of SP-D reached 651.02 ± 56.31 ng/mL, ~4.4-fold higher than that of SP-W (146.59 ± 19.12 ng/mL), indicating markedly enhanced absorption. Furthermore, area under the curve (AUC) values revealed a 3.2-fold increase in total systemic exposure for the SP-D group (3881.31 ± 557.66 ng/mL/h) versus the SP-W group (1210.09 ± 133.08 ng/mL/h), confirming superior bioavailability.

These results indicate that embedding the solid dispersion system in a dairy matrix improves the rate and extent of spinosin absorption, likely by increasing its solubility and prolonging gastrointestinal residence [65]. The extended T_max_ and elevated AUC_0–48h_ values are consistent with a controlled-release effect and an enhanced absorption window. Thus, this formulation strategy may provide a practical and scalable approach for improving the oral delivery of poorly soluble natural compounds [66].

## 4. Discussion

In this study, a spinosin solid-dispersion-based functional dairy beverage was successfully developed and optimised, and its sleep-promoting effect was confirmed in a mouse model. These results confirm that solid dispersion technology effectively improves spinosin’s solubility and bioavailability, enhancing its physiological activity, and integrating it into a widely accepted food carrier provides a novel and feasible approach for sleep disorder intervention.

The solvent melt method proved effective in overcoming spinosin’s poor water solubility. Orthogonal experiments identified optimal parameters that significantly increased apparent solubility, aligning with Zhai et al. [34], who reported that solid dispersion technology enhances the dissolution of poorly soluble traditional Chinese medicine components. Importantly, the present study revealed the impact of particle size on formulation performance. In vitro dissolution experiments showed that reducing the redispersed solid dispersion particle size from 206 to 95 nm markedly accelerated the drug’s release rate and extent. Cellular uptake experiments provided mechanistic insight: smaller particles produced stronger fluorescence signals in Caco-2 cells, indicating easier uptake. This cellular evidence supports the enhanced in vivo efficacy and aligns with Neda et al. [26], indicating that nanoparticles are more readily internalised by biological systems. Overall, nanoscale solid dispersions not only increase dissolution via greater surface area but also improve transmembrane transport, likely contributing to enhanced oral bioavailability.

Following successful solubility enhancement, the dispersion was integrated into a stable functional dairy beverage. Formula optimisation showed that CMC-Na content and solid dispersion amount were the most significant factors affecting the system’s physical stability. An appropriate CMC-Na level inhibited particle aggregation and sedimentation through steric hindrance and electrostatic repulsion, whereas excessive amounts induced bridging flocculation, compromising stability, consistent with the findings of Li et al. [29] in other nano-dispersion systems. The optimised formulation maintained high spinosin loading while ensuring physical stability, demonstrating the feasibility of integrating high-tech nano-preparations into conventional food matrices.

Mechanistically [22,67], spinosin may modulate sleep–wake neural circuits, including GABAergic neurotransmission and orexinergic systems. The observed sleep-promoting effects of the solid dispersion and functional dairy beverage suggest the involvement of these pathways; however, the absence of direct biochemical or neurophysiological measurements is a limitation of the present study. Future research should investigate mechanisms by quantifying neurotransmitters, such as GABA, glutamate and 5-fydroxytryptamine, and assessing expression of sleep-related receptors.

From an application perspective, this study offers a model for addressing the solubility issues of numerous natural active ingredients. With the public’s growing concern regarding the potential side effects of conventional sleeping pills and the increasing demand for safe, natural alternatives, functional products combining food carriers with advanced formulation technologies represent a promising direction in the health industry.

Notably, this study still has certain limitations. First, further work is required to evaluate the long-term physical and chemical stability of spinosin solid dispersions in dairy matrices, including in the developed functional beverage system. Second, the underlying mechanisms of the spinosin solid-dispersion-based functional dairy beverage’s sleep-promoting effects require further study. Finally, prior to human application, the safety and efficacy of the system must be confirmed in higher-order animal models.

## 5. Conclusions

In this study, spinosin–poloxamer 188 solid dispersions were successfully prepared via the solvent fusion method, effectively overcoming spinosin’s poor water solubility. Optimised parameters (a drug-to-carrier mass ratio of 1:2, melting at 70 °C, stirring at 200 rpm and cooling at −15 °C) markedly enhanced apparent solubility and the in vitro dissolution rate. Reducing the redispersed particle size further accelerated the drug release and improved cellular uptake efficiency. PXRD analysis confirmed that spinosin transformed from a crystalline to an amorphous state in the solid dispersion system, qualitatively explaining the solubility increase. A functional dairy beverage with stable physical properties was developed through secondary orthogonal experiments. The optimised formula comprised 40% solid dispersion, 20% milk, 5% white sugar and 0.3% CMC-Na. Animal experiments showed that this functional dairy beverage significantly reduced spontaneous activity in mice, increased sleep induction to 60% under a subthreshold pentobarbital dose and ameliorated ethanol-induced memory impairment. Pharmacokinetic results further validated the superior absorption efficiency and in vivo exposure of spinosin delivered via the solid-dispersion-based functional dairy beverage, reinforcing its translational potential in improving oral bioavailability.

Beyond validating the physicochemical feasibility and sleep-promoting efficacy of the spinosin solid dispersion, this study provides meaningful insights into its translational potential. The preparation process, combining solvent-fusion-based dispersion with conventional dairy manufacturing techniques, uses food-grade excipients and scalable operations, demonstrating compatibility with industrial-scale production. Given growing consumer demand for natural sleep-supporting products, the developed functional dairy beverage shows promise for future human applications. Nevertheless, further research is needed to bridge preclinical effectiveness and real-world use, including comprehensive stability, bioavailability and human safety assessments prior to commercialisation.

## Figures and Tables

**Table 1:** Single-factor experiment conditions for preparing spinosin solid dispersions.

No.	Mass Ratio (%)	Melting Temperature (°C)	Stirring Speed (rpm/min)	Cooling Temperature (°C)
1	1:1	40	25	−20
2	1:2	50	50	−15
3	1:3	60	100	−10
4	1:4	70	150	−5
5	1:5	80	200	0
6	1:6	90	250	5

Mass ratio: spinosin–poloxamer 188 mass ratio.

**Table 2:** Orthogonal experiment design and level settings for spinosin solid dispersion preparation.

Level	A	B	C	D
	Mass Ratio (%)	Melting Temperature (°C)	Stirring Speed (rpm/min)	Cooling Temperature (°C)
1	1:1	60	100	−5
2	1:2	70	150	−10
3	1:3	80	200	−15

Mass ratio: spinosin–poloxamer 188 mass ratio.

**Table 3:** Single-factor experiment for preparation of the spinosin solid dispersion functional dairy beverage.

No.	Spinosin Solid Dispersion (%)	Milk Volume (mL)	White Granulated Sugar Mass Fraction (%)	CMC-Na Mass Fraction (%)
1	20	20	1	0.05
2	30	30	2.5	0.1
3	40	40	5	0.2
4	50	50	7.5	0.3
5	60	60	10	0.4
6	70	70	12.5	0.5

CMC-Na: sodium carboxymethyl cellulose.

**Table 4:** Orthogonal experiment and level design for the spinosin solid-dispersion-based functional dairy beverage.

Level	A	B	C	D
	Spinosin Solid Dispersion (%)	Milk Volume (mL)	White Granulated Sugar Mass Fraction (%)	CMC-Na Mass Fraction (%)
1	30	20	2.5	0.1
2	40	30	5	0.2
3	50	40	7.5	0.3

CMC-Na: sodium carboxymethyl cellulose.

**Table 5:** Results of orthogonal experiment design for spinosin solid dispersion preparation.

Indicators	A	B	C	D	Apparent Solubility
1	1	1	1	1	25.62
2	1	2	2	2	28.37
3	1	3	3	3	29.66
4	2	1	2	3	33.51
5	2	2	3	1	38.29
6	2	3	1	2	31.22
7	3	1	3	2	28.37
8	3	2	1	3	29.26
9	3	3	2	1	25.09
K1	83.65	87.50	86.10	89.00	F_0.05_ = 19 F_0.01_ = 99
K2	103.02	95.92	86.97	87.96	
K3	82.72	85.97	96.32	92.43	
k1	27.88	29.17	28.70	29.67	
k2	34.34	31.97	28.99	29.32	
k3	27.57	28.66	32.11	30.81	
R	6.77	3.32	3.41	1.49	

A: Spinosin–poloxamer 188 mass ratio; B: melting temperature; C: stirring speed; D: cooling temperature; response index: apparent solubility.

**Table 6:** Variance analysis of orthogonal experiment results for spinosin solid dispersion preparation.

Factors	Sum of Squares of Deviations	Degrees of Freedom	F-Ratio	F-Critical Value	Significance
A	87.572	2	43.786	24.01	*
B	19.138	2	9.569	5.25	
C	21.403	2	10.702	5.87	
D	3.647	2	1.824	-	

*: Significant difference at *p* < 0.05.

**Table 7:** Orthogonal experiment design and results for preparation of the spinosin solid-dispersion-based functional dairy beverage.

Indicators	A	B	C	D	Centrifugal Precipitation Rate
1	1	1	1	1	7.35
2	1	2	2	2	6.22
3	1	3	3	3	5.53
4	2	1	2	3	2.05
5	2	2	3	1	5.91
6	2	3	1	2	4.66
7	3	1	3	2	6.21
8	3	2	1	3	4.03
9	3	3	2	1	6.92
K1	19.10	15.61	16.04	20.18	F_0.05_ = 19 F_0.01_ = 99
K2	12.62	16.16	15.19	17.09	
K3	17.16	17.11	17.65	11.61	
k1	6.37	5.20	5.35	6.73	
k2	4.21	5.39	5.06	5.70	
k3	5.72	5.70	5.88	3.87	
R	2.16	0.50	0.82	2.86	

A: Spinosin solid dispersion mass fraction; B: milk volume; C: white granulated sugar mass fraction; D: sodium carboxymethyl cellulose mass fraction; response index: centrifugal precipitation rate.

**Table 8:** Variance analysis of orthogonal experiment results for preparation of the spinosin solid-dispersion-based functional dairy beverage.

Factors	Sum of Squares of Deviations	Degrees of Freedom	F-Ratio	F-Critical Value	Significance
A	7.374	2	3.687	19.21	*
B	0.384	2	0.192	-	
C	1.041	2	0.520	2.71	
D	12.558	2	6.279	32.71	*

*: Significant difference at *p* < 0.05.

**Table 9:** Evaluation of the spinosin solid-dispersion-based preparations’ sleep-promoting effects in Kunming mice (*n* = 20).

Group	Dosage (mg/kg)	Number of Mice	Number of Autonomous Activities (Times/10 min)	Number of Sleeping Animals	Incubation Period (s)	Number of Errors Within 5 min
A	0	20	315.62 ± 19.22	0	35.66 ± 5.02	6.58 ± 1.01
B	10	20	195.96 ± 29.03	12	192.51 ± 3.99	1.69 ± 0.65
C	10	20	289.31 ± 31.58	2	46.32 ± 4.80	5.33 ± 1.25
D	0	20	308.54 ± 17.49	0	37.24 ± 5.81	6.32 ± 0.78

A: Control group (distilled water); B: group receiving the spinosin solid-dispersion-based functional dairy beverage; C: group receiving an aqueous spinosin solution prepared with distilled water; D: group receiving the pure milk drink without spinosin.

**Table 10:** Mean pharmacokinetic parameters of spinosin following oral administration of a spinosin water suspension (SP-W) and a spinosin solid-dispersion-based dairy suspension (SP-D) in rats (*n* = 6).

Parameters	SP-W	SP-D
T_1/2α_ (h)	3.27 ± 0.33	7.19 ± 0.50
T_1/2β_ (h)	8.06 ± 0.91	19.73 ± 1.82
C_max_ (ng/mL)	146.59 ± 19.12	651.02 ± 56.31
AUC_0–48h_ (ng/mL/h)	1210.09 ± 133.08	3881.31 ± 557.66

T_1/2α_: distribution half-life; T_1/2β_: elimination half-life; C_max_: maximum plasma concentration; AUC: area under the curve.

## Data Availability

The data presented in this study are available on request from the corresponding author due to privacy concerns.

## Abbreviations

The following abbreviations are used in this manuscript:

SD	Sprague-Dawley
DLS	Dynamic light scattering
CMC-Na	Sodium carboxymethyl cellulose
GABA	Gamma-aminobutyric acid
KM	Kunming
NREM	Non-rapid eye movement
SIF	Simulated intestinal fluid

## References

[1] Jiayin L., Kanghao C., Ta W.I., Fen B.W. (2025). The effectiveness of the Chinese Five-Element Music intervention on older adults with depression and anxiety disorder: A systematic review and meta-analysis. Medicine.

[2] Morin C.M., Jarrin D.C. (2022). Epidemiology of Insomnia Prevalence, Course, Risk Factors, and Public Health Burden. Sleep Med. Clin..

[3] Meng W., Mingqiang W., Junyang Z., Bin P., Qiong W., Chao W. (2025). Synergistic acupuncture and neuromodulation for chronic insomnia: A structured narrative review with systematic search and future directions. Front. Neurol..

[4] Sivertsen B., Hysing M., Harvey A.G., Petrie K.J. (2021). The Epidemiology of Insomnia and Sleep Duration Across Mental and Physical Health: The SHoT Study. Front. Psychol..

[5] Sukumaran L., Scheuermaier K., Sabin C.A., Chandiwana N., Olivé F.X.G., Schantz M.V., Rae D.E., Winston A. (2025). Understanding and managing disordered sleep in people with HIV. Lancet HIV.

[6] Lee J., Hong Y., Lee W. (2021). Prevalence of Insomnia in Various Industries and Associated Demographic Factors in Night-Shift Workers Using Workers’ Specific Health Examination Data. Int. J. Environ. Res. Public Health.

[7] Huang J., Tang A., Li Q., Chen F., Ye S., Song L., Qiu P. (2025). Network analysis of sleep disorders, depression and anxiety symptoms among community older adults. BMC Geriatr..

[8] Xia J., Chen C., Lu X., Zhang T., Wang T., Wang Q., Zhou Q. (2025). Artificial intelligence-oriented predictive model for the risk of postpartum depression: A systematic review. Front. Public Health.

[9] Gong L., Xie J.-B., Luo Y., Qiu Z.-D., Liu J.-R., Mei N.-J., Chen Z.-Y., Wang F.-L., Huang Y., Guo J. (2023). Research progress of quality control for the seed of *Ziziphus jujuba* var. spinosa (Bunge) Hu ex H.F. Chow (Suan-Zao-Ren) and its proprietary Chinese medicines. J. Ethnopharmacol..

[10] Luo H., Sun S.J., Wang Y., Wang Y.L. (2020). Revealing the sedative-hypnotic effect of the extracts of herb pair Semen Ziziphi spinosae and Radix Polygalae and related mechanisms through experiments and metabolomics approach. BMC Complement. Med. Ther..

[11] Bi F., Wang Z., Guo Y., Xia M., Zhu X., Qiao W. (2024). A Combination of Magnoflorine and Spinosin Improves the Antidepressant effects on CUMS Mouse Model. Curr. Drug Metab..

[12] Zhou C., Chen H., Li W., Yang L., Zeng L., Bian Y. (2025). Mechanism of *Sour jujube* Kernel-five-flavour Berry in the Treatment of Insomnia based on Network Pharmacology and Molecular Docking. Curr. Pharm. Des..

[13] Fang X.S., Hao J.F., Zhou H.Y., Zhu L.X., Wang J.H., Song F.Q. (2010). Pharmacological studies on the sedative-hypnotic effect of Semen Ziziphi spinosae (Suanzaoren) and Radix et Rhizoma Salviae miltiorrhizae (Danshen) extracts and the synergistic effect of their combinations. Phytomedicine.

[14] Bian Z.H., Zhang W.M., Tang J.Y., Fei Q.Q., Hu M.M., Chen X.W., Su L.L., Fei C.H., Ji D., Mao C.Q. (2021). Mechanisms Underlying the Action of Ziziphi Spinosae Semen in the Treatment of Insomnia: A Study Involving Network Pharmacology and Experimental Validation. Front. Pharmacol..

[15] Zhang M., Liu J.R., Zhang Y.Q., Xie J.B. (2022). Ziziphi Spinosae Semen: A Natural Herb Resource for Treating Neurological Disorders. Curr. Top. Med. Chem..

[16] Xiaolan K., Ganshu S., Ting M., Wanna C., Jingjing Z., Bo L., Fangfang X. (2022). The pharmacology, pharmacokinetics, and toxicity of spinosin: A mini review. Front. Pharmacol..

[17] He S., Li M., Chen Y., Liu Q. (2025). Environmental and biological factors influence flavonoid and saponin contents in Ziziphi Spinosae Semen in the taihang mountains. Ind. Crops Prod..

[18] Wang L.-E., Cui X.-Y., Cui S.-Y., Cao J.-X., Zhang J., Zhang Y.-H., Zhang Q.-Y., Bai Y.-J., Zhao Y.-Y. (2010). Potentiating effect of spinosin, a C-glycoside flavonoid of Semen Ziziphi spinosae, on pentobarbital-induced sleep may be related to postsynaptic 5-HT 1A receptors. Phytomedicine.

[19] Il K.W., Tian Z.B., Yan Z.H., Hyun L.J., Keun S.J., Hee W.M. (2014). Quantitative and pattern recognition analyses of magnoflorine, spinosin, 6‴-feruloyl spinosin and jujuboside A by HPLC in Zizyphi Semen. Arch. Pharmacal Res..

[20] Shin K.H., Lee C.K., Woo W.S., Kang S.S. (1978). Sedative action of spinosin. Arch. Pharmacal Res..

[21] Moon K.M., Hwang Y.-H., Yang J.-H., Ma J.Y., Lee B. (2019). Spinosin is a flavonoid in the seed of *Ziziphus jujuba* that prevents skin pigmentation in a human skin model. J. Funct. Foods.

[22] Zhao W., Zhang H., Li L., Zhang J., Chu L. (2025). Spinosin enhances non-rapid eye movement sleep and alters c-Fos expression in sleep–wake regulatory brain regions in mice. Sleep Breath..

[23] Zhang J.P., Liao D.Q., Li L., Chu L. (2020). Reduced c-Fos expression in orexin neurons of the lateral hypothalamic area and the locus coeruleus following injection of spinosin into mice. Folia Morphol..

[24] Ying-Jie D., Ning-Hua J., Liang-Hui Z., Xi T., Xi F., Min-Qiu L., Zhi-Yi X., Rong L., Lin-Zi L., Bo L. (2021). Soporific effect of modified Suanzaoren Decoction on mice models of insomnia by regulating Orexin-A and HPA axis homeostasis. Biomed. Pharmacother..

[25] Mingzhen L., Wenxia Y., Yao X., Zhijie D., Wei H., Fan Z., Xinke Z., Min L. (2021). Resveratrol-modified mesoporous silica nanoparticle for tumor-targeted therapy of gastric cancer. Bioengineered.

[26] Neda N., Parvin Z.-M., Hamed H., Younes P.-S., Hadi V. (2017). Development, In Vitro Characterization, Antitumor and Aerosol Performance Evaluation of Respirable Prepared by Self-nanoemulsification Method. Drug Res..

[27] Rassu G., Porcu E.P., Fancello S., Obinu A., Senes N., Galleri G., Migheli R., Gavini E., Giunchedi P. (2018). Intranasal Delivery of Genistein-Loaded Nanoparticles as a Potential Preventive System against Neurodegenerative Disorders. Pharmaceutics.

[28] Budiman A., Ivana H., Huang K.A., Huang S.A., Nadhira M.S., Rusdin A., Aulifa D.L. (2025). Biocompatible Natural Polymer-Based Amorphous Solid Dispersion System Improving Drug Physicochemical Properties, Stability, and Efficacy. Polymers.

[29] Li Y., Wang Q., Pan J., Zhao X., Zhan J., Xu X., Zhang M., Wang C., Cui H. (2025). Fabrication and Characterization of a Novel Solid Nano-Dispersion of Emamectin Benzoate with High Dispersibility and Wettability. Nanomaterials.

[30] Kyosuke S., Kohsaku K., Masafumi F., Michinori O., Yohei N., Maki M., Takuya F. (2021). Relevance of Liquid-Liquid Phase Separation of Supersaturated Solution in Oral Absorption of Albendazole from Amorphous Solid Dispersions. Pharmaceutics.

[31] Halder S., Ahmed F., Shuma M.L., Azad M.A.K., Kabir E.R. (2020). Impact of drying on dissolution behavior of carvedilol-loaded sustained release solid dispersion: Development and characterization. Heliyon.

[32] Kaushika P., Shreeraj S., Jaymin P. (2022). Solid dispersion technology as a formulation strategy for the fabrication of modified release dosage forms: A comprehensive review. Daru J. Fac. Pharm. Tehran Univ. Med. Sci..

[33] Wang J., Shi C., Jiao W., Wang X., Sang Y. (2024). Study on the neuroprotective and sedative effects of the combination of jujuboside B and spinosin from Ziziphi Spinosae Semen. J. Funct. Foods.

[34] Zhai X., Li C., Lenon G.B., Xue C.C.L., Li W. (2016). Preparation and characterisation of solid dispersions of tanshinone IIA, cryptotanshinone and total tanshinones. Asian J. Pharm. Sci..

[35] Shahverdi F., Khodaverdi E., Movaffagh J., Mehrjardi S.T., Kamali H., Nokhodchi A. (2025). Lycopene-Carrier Solid Dispersion loaded Lipid Liquid Crystal Nanoparticle: In vitro Evaluation and in vivo Wound Healing Effects. Pharm. Dev. Technol..

[36] Ji H., Shi X. (2025). Hydroxypropyl methylcellulose E5 with molecular energy-enhancement ability contributes to improving wettability, drug delivery and taste masking effect for curcumin solid dispersions. Int. J. Biol. Macromol..

[37] Chen J., Yao Y. (2023). Phytoglycogen to Enhance the Solubility and in-vitro Permeation of Resveratrol. Food Biophys..

[38] Han G., Wang B., Jia M., Ding S., Qiu W., Mi Y., Mi Z., Qin Y., Zhu W., Liu X. (2022). Optimization and evaluation of resveratrol amorphous solid dispersions with a novel polymeric system. Math. Biosci. Eng. MBE.

[39] Cao Y., Teng J., Selbo J. (2017). Amorphous Solid Dispersion of Epigallocatechin Gallate for Enhanced Physical Stability and Controlled Release. Pharmaceuticals.

[40] Yasuda J., Yoshizaki T., Yamamoto K., Yoshino M., Ota M., Kawahara T., Kamei A. (2019). Association of Frequency of Milk or Dairy Product Consumption with Subjective Sleep Quality during Training Periods in Japanese Elite Athletes: A Cross-Sectional Study. J. Nutr. Sci. Vitaminol..

[41] Oboh M., Elhassan E., Koorbanally N.A., Govender L., Siwela M., Govender T., Mkhwanazi B.N. (2025). Characterisation and In Vitro Drug Release Profiles of Oleanolic Acid- and Asiatic Acid-Loaded Solid Lipid Nanoparticles (SLNs) for Oral Administration. Pharmaceutics.

[42] Brough C., Williams R.O. (2013). Amorphous solid dispersions and nano-crystal technologies for poorly water-soluble drug delivery. Int. J. Pharm..

[43] Leuner C., Dressman J. (2000). Improving drug solubility for oral delivery using solid dispersions. Eur. J. Pharm. Biopharm..

[44] Kahrass H., Pietschmann I., Mertz M. (2024). Why Do I Choose an Animal Model or an Alternative Method in Basic and Preclinical Biomedical Research? A Spectrum of Ethically Relevant Reasons and Their Evaluation. Animals.

[45] Wang L.E., Bai Y.J., Shi X.R., Cui X.Y., Cui S.Y., Zhang F., Zhang Q.Y., Zhao Y.Y., Zhang Y.H. (2008). Spinosin, a C-glycoside flavonoid from semen Zizhiphi Spinozae, potentiated pentobarbital-induced sleep via the serotonergic system. Pharmacol. Biochem. Behav..

[46] Li Y.J., Liang X.M., Xiao H.B., Bi K.S. (2003). Determination of spinosin in rat plasma by reversed-phase high-performance chromatography after oral administration of Suanzaoren decoction. J. Chromatogr. B-Anal. Technol. Biomed. Life Sci..

[47] Li Y.-J., Dai Y.-H., Yu Y.-L., Li Y., Deng Y.-L. (2007). Pharmacokinetics and Tissue Distribution of Spinosin after Intravenous Administration in Rats. Yakugaku Zasshi.

[48] Ali W., Williams A.C., Rawlinson C.F. (2010). Stochiometrically governed molecular interactions in drug: Poloxamer solid dispersions. Int. J. Pharm..

[49] Dugar R.P., Gajera B.Y., Dave R.H. (2016). Fusion Method for Solubility and Dissolution Rate Enhancement of Ibuprofen Using Block Copolymer Poloxamer 407. AAPS PharmSciTech.

[50] Wassvik C.M., Holmén A.G., Bergström C.A.S., Zamora I., Artursson P. (2006). Contribution of solid-state properties to the aqueous solubility of drugs. Eur. J. Pharm. Sci..

[51] Zhefei G., Ming L., Yongcheng L., Huishi P., Ling L., Xu L., Chuanbin W. (2014). The utilization of drug-polymer interactions for improving the chemical stability of hot-melt extruded solid dispersions. J. Pharm. Pharmacol..

[52] Moore T., Croy S., Mallapragada S., Pandit N. (2000). Experimental investigation and mathematical modeling of Pluronic F127 gel dissolution: Drug release in stirred systems. J. Control. Release Off. J. Control. Release Soc..

[53] Ji Y., Paus R., Prudic A., Lübbert C., Sadowski G. (2015). A Novel Approach for Analyzing the Dissolution Mechanism of Solid Dispersions. Pharm. Res..

[54] Kolisnyk T., Mohylyuk V., Fil N., Bickerstaff E., Li S., Jones D.S., Andrews G.P. (2024). High drug-loaded amorphous solid dispersions of a poor glass forming drug: The impact of polymer type and cooling rate on amorphous drug behaviour. Int. J. Pharm..

[55] Rahul L., Krishna K.N.S., Raj S. (2023). Understanding the Effect of Nucleation in Amorphous Solid Dispersions through Time-Temperature Transformation. Mol. Pharm..

[56] Ahmed L.M., Mohamed F.A., Elfaham T.H. (2025). Nanocrystals as a promising approach for enhancing solubility and dissolution of etoricoxib using Box–Behnken design. Sci. Rep..

[57] Wenjun S., Yang T., Zengming W., Hui Z., Aiping Z. (2022). The Study of Cyclosporin A Nanocrystals Uptake and Transport across an Intestinal Epithelial Cell Model. Polymers.

[58] Andar A.U., Hood R.R., Vreeland W.N., Devoe D.L., Swaan P.W. (2014). Microfluidic Preparation of Liposomes to Determine Particle Size Influence on Cellular Uptake Mechanisms. Pharm. Res..

[59] Sharma A., Jain C.P., Tanwar Y.S. (2013). Preparation and characterization of solid dispersions of carvedilol with poloxamer 188. J. Chil. Chem. Soc..

[60] Antonopoulou E., Rohmann-Shaw C.F., Sykes T.C., Cayre O.J., Hunter T.N., Jimack P.K. (2018). Numerical and experimental analysis of the sedimentation of spherical colloidal suspensions under centrifugal force. Phys. Fluids.

[61] Lee H. (2024). Hydrodynamics and Aggregation of Nanoparticles with Protein Corona: Effects of Protein Concentration and Ionic Strength (Small 51/2024). Small.

[62] Liu N., Chen Q., Li G., Zhu Z., Yi J., Li C., Chen X., Wang Y. (2018). Properties and Stability of Perilla Seed Protein-Stabilized Oil-in-Water Emulsions: Influence of Protein Concentration, pH, NaCl Concentration and Thermal Treatment. Molecules.

[63] Canales M.A.M., Hidalgo M.E., Risso P.H. (2010). Colloidal Stability of Bovine Calcium Caseinate Suspensions. Effect of Protein Concentration and the Presence of Sucrose and Lactose. J. Chem. Eng. Data ACS J. Data.

[64] Yun T., Lee S., Yun S., Cho D., Bang K., Kim K. (2024). Investigation of Stabilized Amorphous Solid Dispersions to Improve Oral Olaparib Absorption. Pharmaceutics.

[65] Schver G.C.R.M., Sun D.D., Costa S.P.M., Silva K.E.R., Oliveira J.F., Rolim L.A., Albuquerque M.C.P.d.A., Aires A.d.L., Lima M.d.C.A., Pitta I.R. (2018). Solid dispersions to enhance the delivery of a potential drug candidate LPSF/FZ4 for the treatment of schistosomiasis. Eur. J. Pharm. Sci..

[66] Ha E.-S., Choi D.H., Baek I.-h., Park H., Kim M.-S. (2021). Enhanced Oral Bioavailability of Resveratrol by Using Neutralized Eudragit E Solid Dispersion Prepared via Spray Drying. Antioxidants.

[67] Du Y., Wang J., Jiang L., Li J., Li J., Ren C., Yan T., Jia Y., He B. (2023). Screening the components in multi-biological samples and the comparative pharmacokinetic study in healthy and depression model rats of Suan-Zao-Ren decoction combined with a network pharmacology. J. Ethnopharmacol..

